# Outcomes of Wavefront-Optimized Laser-Assisted *In-Situ* Keratomileusis and Photorefractive Keratectomy for correction of Myopia and Myopic Astigmatism over One Year Follow-Up

**DOI:** 10.2174/1874364101812010256

**Published:** 2018-09-26

**Authors:** Mohammad M. Shehadeh, Mohammad T. Akkawi, Ammar A. Aghbar, Muna T. Musmar, Malak N Khabbas, Marah F Kharouf, Liana Al-Labadi

**Affiliations:** 1 Faculty of Medicine and Health Sciences, An-Najah National University Hospital, An-Najah National University, Nablus, Palestine; 2 Vardinoyannion Eye Institute of Crete (VEIC), Faculty of Medicine, University of Crete, Heraklion, Crete, Greece

**Keywords:** Laser-assisted *in-situ* keratomileusis, Photorefractive keratectomy, Wavefront-optimized, Outcomes, Safety, Efficacy, Predictability

## Abstract

**Background:**

Laser corneal refractive surgery suits, technology and nomograms are improving with time. This may improve the refractive and visual outcomes of the patients.

**Objectives:**

To evaluate the safety, efficacy, stability, and predictability of wavefront-optimized photorefractive keratectomy and Laser-assisted *in-situ* keratomileusis in patients with myopia and myopic astigmatism over 1-year using WaveLight^®^ EX500 Excimer Laser machine.

**Methods:**

In this prospective cohort study, refractive and visual outcomes in 596 eyes (365 patients), either having myopia or myopic astigmatism were assessed. Patients were divided into Two groups: 1) Patients who underwent PRK (53 eyes have myopia and 217 eyes have myopic astigmatism), 2) Patients who underwent LASIK (53 eyes have myopia and 273 eyes have myopic astigmatism).

**Results:**

At 12 months postoperatively 94.3% of the myopic patients reached their preoperative best corrected distance visual acuity at the final one year follow up visit post PRK and LASIK. In patients with myopic astigmatism who underwent LASIK and PRK, 95.2%, and 96.3% of the patients reached their preoperative best corrected distance visual acuity at the final one year follow up visit post LASIK and PRK, respectively. The efficacy and safety indices were 1.00 or more for all groups with no eye lost any line of best corrected distance visual acuity.

**Conclusion:**

Our study results confirm the excellent efficacy, safety, good predictability and stability of myopia / myopic astigmatism correction by either wavefront- optimized LASIK or PRK over 1-year follow-up without significant differences between them using the WaveLight^®^ EX500 excimer laser system.

## 1 INTRODUCTION

Refractive errors, including myopia, hyperopia and astigmatism are common treatable problems. There are many methods for correction of refractive errors; simple non-invasive methods such as spectacles and contact lenses are used commonly. However, Spectacles and contact lenses usually affect the life style of patients, especially those who are physically active and wearing spectacles and carrying contact lens solution may be a hindrance to them. Corneal refractive surgeries have become increasingly common for long-term correction of refractive error for achieving spectacle and contact lenses independency. Laser-Assisted *in-Situ* Keratomileusis (LASIK) and Photorefractive Keratectomy (PRK) procedures work on reshaping the cornea by excimer laser to modify its refractive power [1].

Many ophthalmologists prefer Lasik over PRK mainly because of the speed of visual recovery and the less post-operative pain. It also avoids complications of PRK such as corneal haze when correcting high degrees of refractive errors, and the risk of delayed epithelial healing and/or infection. On the other hand, other refractive surgeons prefer PRK over LASIK. They claim that Lasik has its own complications, which are related to the flap creation, such as free or incomplete flap, epithelial ingrowth and diffuse lamellar keratitis [2, 3]. However, both procedures proved to be safe and effective in treating refractive errors, and the choice of the procedure should be customized according to each case.

Conventional laser treatment profiles tend to induce higher-order aberrations because they have small blend zones and create a more oblate corneal shape [4]. Wavefront-optimized laser ablation profile minimizes the induction of higher-order aberrations by increasing the pulses over the peripheral cornea to follow the shape of the curved cornea which improves the postoperative corneal shape. This ablation profile does not address the preoperative higher-order aberration [5]. Wavefront-guided laser ablation profile attempts to treat both lower-order aberrations (myopia, hyperopia and/or astigmatism) and higher-order aberrations using the information from a wavefront-sensing aberrometer; however, many studies have found that the majority of patients do not have significant preoperative higher-order aberrations [6].

In this study, we present the outcomes of wavefront- optimized PRK and Lasik for myopia and myopic astigmatism over one year follow up.

## 2 MATERIALS AND METHODS

In this prospective cohort study, 596 eyes (365 patients). Patients were divided into two groups: 1) Patients who underwent PRK (53 eyes have myopia and 217 eyes have myopic astigmatism), 2) Patients who underwent LASIK (53 eyes have myopia and 273 eyes have myopic astigmatism). All were performed by the same cornea and refractive surgeon (MMSH) at An-Najah National University Hospital in Nablus, Palestine; in the period between May 2014 and May 2017 using refractive surgery suite (WaveLight^®^ EX500 Excimer Laser; Alcon Laboratories, Ft Worth, TX, USA). Prior approval by ethical committee of An-Najah National University -Faculty of Medicine and Health sciences was obtained according to the Declaration of Helsinki. The participants were informed about the purpose of the study and a written informed consent was signed by them before inclusion.

Inclusion criteria were age of 18 years or older patients, a postoperative follow-up of at least 12 months and preoperative stable refraction for a least one-year, normal corneal tomography, refractive error of myopia or myopic astigmatism (simple or compound), and expected postoperative residual stromal bed more than 300μm or 55% of the thinnest corneal thickness. Exclusion criteria were age under 18, refractive error of hyperopia or hyperopic astigmatism, previous ocular surgeries, and corneal, retinal, or uveal diseases. Also, any patient with systemic disease that may affect refractive status or post-operative healing process (*e.g.* diabetes mellitus) was excluded.

The pre-operative examination included the measurement of Uncorrected Distance Visual Acuity (UDVA) and best Corrected Distance Visual Acuity (CDVA) using a Snellen chart (decimals), the manifest spherical, cylindrical refraction and Manifest Refraction Spherical Equivalent (MRSE). The corneal tomography was obtained using Scheimpflug topography (Pentacam HR; Oculus, Wetzlar, Germany) showing parameters of flat keratometry (K1), steep keratometry (K2), mean keratometry (K m), maximal keratometry (K max), and the thinnest location’s corneal thickness.

Full ophthalmic examination included slitlamp biomicroscopy for anterior and posterior segments evaluation, Goldmann applanation tonometry, pupillometry and Schirmer test. Postoperative follow-up examinations were conducted at 1 day, 1 week, 1 month, 3 months, 6 months, and 1-year intervals.

The selection of PRK or LASIK was determined based on the preoperative tomography, pachymetry, subsequent assumed risk of ectasia, patient’s job and hobbies and patient’s preference.

All operations were performed under topical anesthesia with proparacaine hydrochloride 0.5% by one surgeon (MMSH). The WaveLight^®^ EX500 Excimer Laser refractive surgery suit. Moria One Use- plus SBK mechanical microkeratome (Moria, Antony, France) were used for flap creation in Lasik and Amoils corneal brush (Innovative Excimer Solutions, Toronto, Canada) was used for epithelial removal in PRK. For all patients, the optical zone ablated was 6.5 mm with a total ablation zone of between 7.1 to 9.0 mm. The total ablation zone was calculated based on preoperative low mesopic pupil size. Wavefront-optimized treatment profiles were used for all patients with emmetropia being the target refraction.

For PRK, after the mechanical removal of the corneal epithelium, surface laser ablation was performed. A sponge soaked in mitomycin- C (MMC) 0.02% was placed on the ablated stromal bed (for 10 seconds per 1.0 D correction). Then, the stroma was irrigated with 30 cc balanced salt solution. At the end of the procedure, a bandage contact lens was placed. The postoperative treatment included Gatifloxacin Eye Drops (E/D) four times daily for a week, Dexamethasone Sodium Phosphate E/D four times daily tapered over one month and preservative-free lubricant eye drops for at least 3 months. To reduce post-operative pain, combined Paracetamol and Tramadol 325mg/37.5mg tablets were used every 8 hours for the first two days. The bandage contact lens was removed after complete epithelial healing at the 1-week follow up visit.

For LASIK, after topical anesthesia and marking the corneal surface, the One Use- Plus SBK microkeratome was utilized to make a nasally hinged corneal flap of 90 *μ*m thickness. Laser ablation was done, and the flap was repositioned. Irrigation by balanced salt solution was performed. Milking the flap gently by wet spear was done to enhance the adherence of the flap. The surgeon waited 2 minutes before removing the speculum to insure adequate time for flap adherence. Post-operative regimen included Gatifloxacin E/D four times daily for one week, Dexamethasone Sodium Phosphate E/D four times daily for 2 weeks and preservative-free lubricant eye drops for at least three months.

## 3 STATISTICAL ANALYSIS

Data were analyzed using the statistical packages for social sciences SPSS (version 22.0). Descriptive statistics were generated for continuous variables and categorical variables. Paired sample T test was used. The chosen level of statistical significance was *P* <0.05.

## 4 RESULTS

Five hundred ninety-six (596) eyes were enrolled in this study. The mean age of all patients was 26.5 years (for LASIK patients was 27.2, and for PRK patients was 25.9). PRK was done in 270 eyes, and LASIK was done in 326 eyes as shown in Table **1**.

### 4.1 Myopic Patients

The mean sphere corrected in PRK patients was -2.20 +/- 0.99 (range from -0.75 to -5.25) but it was higher in Lasik patients -3.34 +/- 2.12 (range from -1.25 to -8.25). One year post operatively, the mean sphere changed to -0.04 +/- 0.03, -0.04 +/- 0.03 in patients who underwent PRK and LASIK respectively (*P*<0.0001).

The mean preoperative UDVA (decimal) in PRK patients was 0.22+/- 0.16 (range from 0.02 to 0.7) which improved to (0.85+/-0.18) on the first day post operatively (*P*<0.0001) and continued to improve till the third month post operatively (1.06 +/- 0.15). No significant change was noticed thereafter. The mean UDVA was 1.02 +/- 0.071 at one-year post-operative visit. On the other hand, the preoperative CDVA was 1.01 which remained stable post operatively; none of the patients lost any line of their CDVA. The UDVA of 94.3% of the myopic patients (50/53 eyes) reached their preoperative CDVA at the final one-year follow up visit. Three eyes did not reach their pre-operative CDVA, where they had 0.9 UDVA at one- year post-operative visit which improved to 1.00 with refraction (MRSE range: -0.25 to -0.50).

For participants who underwent LASIK, the preoperative mean UDVA was 0.13+/- 0.14 (range from 0.02 to 0.6) which improved to 1.01 on the first day post operatively (*P* <0.0001) and remained stable through the whole post-operative visits. The mean UDVA was 1.01 at one year follow up visit. Preoperative CDVA was 1.01+/-0.20 (range from 0.3 to 1.5), which remained stable post operatively; none of the patients lost any line of their CDVA. The UDVA of 94.3% of the myopic patients (50/53 eyes) reached their preoperative CDVA at the final one year follow up visit. The other three eyes that didn’t reach their pre-operative CDVA had UDVA at one year of (0.6, 0.8, 0.9) which improved to 1.00 with refraction (MRSE range: -0.25 to -1.25).

K1, K2, K m, K max and thinnest corneal thickness were obtained using Pentacam as shown in Table **2**.

### 4.2 Myopic Astigmatism Patients

For patients with myopic astigmatism who underwent PRK, the mean pre-operative MRSE value was -2.74 ± 1.35 (range from -0.25 to -7.88) which improved to -0.025 ± 0.0074 one year post operatively (*P*<0.0001). The mean UDVA was 0.20 ± 0.20 (range from 0.01 to 0.8) which improved to 0.74 ± 0.24 (range from 0.2 to 1.2) on the first day post operatively (*P*<0.0001) and continued to improve till the third month post operatively; *i.e.* 1.03 ± 0.12. No significant change was noticed thereafter. Mean UDVA was 1.02 ± 0.09 at one-year post-operative visit. On the other hand, the preoperative CDVA was 1.03 which remained stable post operatively; none of the patients lost any line of their BCVA. The UDVA of 96.3% of the patients (209/217 eyes) reached their preoperative CDVA at the last one-year follow up visit. The UDVA of the other eight eyes that did not reach their pre-operative CDVA had a range from 0.7 to 0.9 at one year which improved to 1.00 with refraction (MRSE range -0.50 to -1.31). In addition, 3.8% (8/209 eyes) showed better UDVA than their pre-operative CDVA.

For patients with myopic astigmatism who underwent LASIK, the mean pre-operative MRSE value was -4.03 ± 1.88 (range from -0.88 to -11.75) which improved to -0.0459 ± 0.019 one year post operatively (*P*<0.0001). The mean UDVA was 0.122 ± 0.138 (range from 0.01 to 0.7) which improved to 1.02 ± 0.19 (range from 0.2 to 1.5) on the first day post operatively (*P*<0.0001) and remained stable through the whole post-operative visits. Mean UDVA was 0.99 +/- 0.12 at one-year post-operative visit. On the other hand, the preoperative CDVA was 1.00±0.11 which remained stable post operatively; none of the patients lost any line of their CDVA. The UDVA of 95.2% of the patients (260/273 eyes) reached their preoperative CDVA at the final one-year follow up visit. The other thirteen eyes that did not reach their pre-operative CDVA had UDVA range from 0.6 to 0.9 at one year that improved to 1.00 with refraction (MRSE range: -0.50 to -1.34). On the other hand, 8.1% (21/260 eyes) showed better UDVA than their pre-operative CDVA.

K1, K2, K m, K max and thinnest corneal thickness were obtained using Pentacam as shown in Table **3**.

**Fig. (1):**
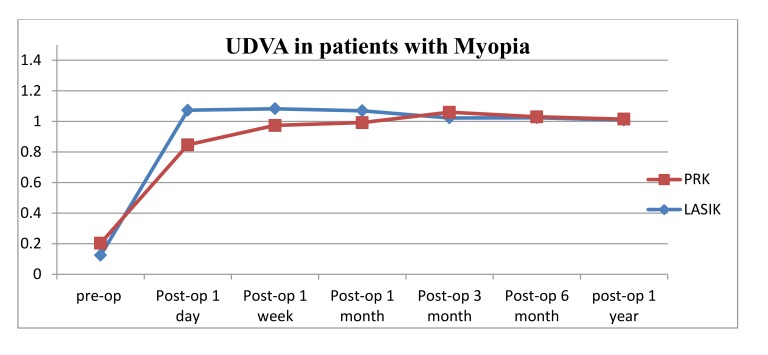
UDVA in patients with myopia underwent LASIK, PRK (pre-operatively to one-year interval).

**Fig. (2):**
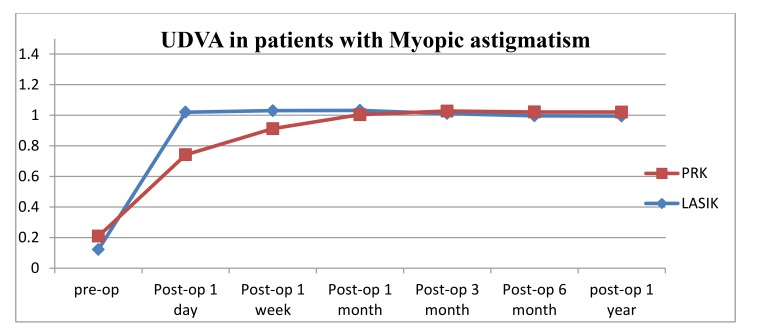
UDVA in patients with myopic astigmatism underwent LASIK, PRK (pre-operatively to one-year interval).

The changes in the mean UDVA pre-operatively to one year post refractive surgeries (LASIK, PRK) for patients with myopia and myopic astigmatism are shown in (Table **4**, Figs. **1** and **2**). For both refractive surgeries (PRK, LASIK), there were significant changes in the UDVA preoperatively to 1-year postoperatively (*P* < 0.001) in both groups of myopia and myopic astigmatism. But the changes in pre-operative best CDVA to the UDVA at 1-year post-operative visit were not significant (*P*>0.05).

There were no serious complications, such as decentered ablation, infection, significant haze formation or LASIK flap-related complications noted in any patient of our study.

The efficacy index (postoperative UDVA/preoperative CDVA) for both PRK and LASIK in myopia and myopic astigmatism is shown in Table **5**.

## 5 DISCUSSION

In this study, we evaluated the efficacy, safety, stability, and predictability of PRK and LASIK in patients with myopia and myopic astigmatism over one-year.

At one year postoperatively, myopic patients achieved UDVA of 1.0 or better in 94.33% and 92.45% of eyes that underwent LASIK and PRK, respectively. Patients with myopic astigmatism achieved UDVA of 1.0 or better in 86.44% and 92.62% of eyes that underwent LASIK and PRK, respectively. To ensure accuracy in evaluating the efficacy of PRK and LASIK, comparison between the pre-operative CDVA and UDVA at one-year post surgery was done, taking into account those participants who have underlying mild amblyopia with a CDVA of less than 1.0. Results showed that 94.3% (50/53 eyes) of the myopic patients reached their preoperative CDVA at the final one year follow up visit post PRK and LASIK. In patients with myopic astigmatism who underwent LASIK and PRK, 95.2% (260/273 eyes) and 96.3% (209/217 eyes) of the patients reached their preoperative CDVA at the final one year follow up visit post LASIK and PRK respectively. This small difference may have been attributed to the higher MRSE in LASIK compared to PRK as shown previously.

Other studies showed results of achieving UDVA of 1.0 or better in 42.6% to 58% of eyes treated by PRK for myopic astigmatism [7-14].

More recent studies have nearly the same or slightly better results and outcomes [15-19]. A study done by Gambato C *et al*. [17] on patients with myopia and myopic astigmatism using wavefront-optimized Surface Ablation with the Allegretto Wave Eye-Q Excimer Laser Platform over 12-month period showed that 94.7% (287/303) of eyes had postoperative UDVA equal or slightly better than pre op CDVA which is nearly the same for our results. Shortt AJ *et al*. [20] showed evidence for superiority of LASIK over PRK for correction of myopia in terms of efficacy and safety but this was not seen in our study as there was no significant difference between the results of two procedures.

The efficacy index (postoperative mean UDVA/preoperative mean CDVA) was nearly 1.00 in all groups as shown previously and the safety index (postoperative CDVA/ preoperative CDVA) was 1.00 or more for all groups with no eye losing any lines of best CDVA, with some patients reported to be gaining one or more lines post-surgery.

Stability of UDVA and refraction was observed in our study between the 3 and 12 months after surgery in both PRK and LASIK, but for patients who underwent LASIK we observed faster improvement of UDVA despite nearly the same outcome for both groups one-year post surgery.

The mean MRSE at one-year post surgery for all patients who underwent LASIK was -0.0418, and -0.02995 for patients who underwent PRK. For those who did not reach 1.0 for both groups, MRSE were in myopic side (-0.25 to -1.00) with a mean of (-0.32) in LASIK patients and (-0.28) in PRK patients. This shows the excellent predictability of PRK and LASIK using WaveLight^®^ EX500 Excimer Laser.

## CONCLUSION

Our study results confirm the excellent efficacy, safety, good predictability and stability myopia / myopic astigmatism correction by either of wavefront- optimized LASIK or PRK over 1-year follow-up without significant differences between them using the WaveLight^®^ EX500 excimer laser system. Outcomes of Lasik and PRK are improving over time. This could be attributed to the improving excimer laser machines ablation profiles, faster ablation, quicker trackers and refinement of nomograms. There is no significant difference in efficacy and safety between PRK and Lasik for the treatment of Myopia and myopic astigmatism. The choice between PRK and Lasik should be taken by studying each case individually.

## Figures and Tables

**Table 1:** Number of eyes that underwent refractive surgeries (PRK, LASIK).

–	**Myopia**	**Myopic Astigmatism**
PRK	53	217
LASIK	53	273

**Table 2:** Pre-operative characteristics of patients with myopia underwent LASIK and PRK.

Pre-Op	Myopic Patients Who Underwent LASIK (53 Patients)			**Myopic Patients Who Underwent PRK** **(53 Patients)**		
	**min**	**max**	mean	**min**	**max**	**mean**
**Age**	19	40	26.9	18	39	24.7
**UDVA**	0.02	0.6	0.13	0.02	0.70	0.22
**CDVA**	0.9	1.2	1.007	0.70	1.20	1.009
**Sphere**	-1.25	-8.5	-3.34	-0.75	-5.25	-2.2
**K1**	40.7	46.6	43.5	40.4	45.40	43.7
**K2**	41.2	47.5	44.2	40.6	46.9	44.4
**Kmean**	41	47.0	44.0	40.5	46.3	44.1
**Kmax**	41.9	47.7	44.5	40.8	47.00	45.1
**Thinnest corneal thickness**	493	611	550.7	487.9	617.0	535.1

**Table 3:** Pre-operative characteristics of patients with myopic astigmatism who underwent LASIK or PRK.

**Pre-op. Characteristics**	**Patients With Myopic Astigmatism Who Underwent LASIK** **(273 Eye)**			**Patients With Myopic Astigmatism Who Underwent** **PRK** **(217 eye)**		
	**Min**	**Max**	**Mean**	**Min**	**Max**	**Mean**
**Age**	18	51	27.3	18	48	27.1
**UDVA**	0.01	0.70	0.12	0.01	0.8	0.20
**CDVA**	0.15	1.20	0.99	0.70	1.20	1.03
**Sphere**	0.00	-10.75	-3.42	0.00	-7.50	-2.23
**Cylinder**	-0.15	-5.25	-1.23	-0.25	-4.75	-1.01
**MRSE**	-0.88	-11.75	-4.03	-0.25	-7.88	-2.74
**K1**	37.5	47.4	43.0	36.7	46.1	43.0
**K2**	34.6	48.9	44.3	39.9	47.5	44.3
**Kmean**	38.1	48.1	43.7	37.0	46.6	43.6
**Kmax**	40.6	49.3	44.9	40.3	48.00	44.7
**Thinnest corneal thickness**	494.8	663.0	546.8	473.8	610.0	532.9

**Table 4:** Change in the mean UDVA from the pre-operative visit to one year post refractive surgeries (LASIK, PRK) for patients with myopia and myopic astigmatism.

	**LASIK** **Myopia**	**PRK** **Myopia**	**LASIK** **Myopic Astigmatism**	**PRK** **Myopic Astigmatism**
**Number of eyes**	53	53	273	217
	mean	mean	mean	mean
**Pre-Op UDVA**	0.13	0.22	0.12	0.20
**Pre-Op CDVA**	1.007	1.009	0.9954	1.039
**UDVA 1 day**	1.0736	0.8472	1.0201	0.7419
**UDVA 1 week**	1.083	0.9736	1.0304	0.9124
**UDVA1 month**	1.0698	0.9925	1.0319	1.0041
**UDVA 3 months**	1.0226	1.0604	1.0110	1.0272
**UDVA 6 months**	1.0245	1.0302	0.9960	1.0217
**UDVA year**	1.0094	1.0151	0.9945	1.0212

**Table 5:** The Efficacy index for both PRK and LASIK in myopic and myopic astigmatism patients.

–	**LASIK** **Myopia**	**PRK** **Myopia**	**LASIK** **Myopic astigmatism**	**PRK** **Myopic astigmatism**
**Post-op 1-year UDVA**	1.0094	1.0151	0.9945	1.0212
**Pre-op best CDVA**	1.007	1.009	0.9954	1.039
**The Efficacy index**	1.0023	1.006	0.999	0.982
